# Postoperative stroke in acute type A aortic dissection: incidence, outcomes, and perioperative risk factors

**DOI:** 10.1186/s12893-024-02499-9

**Published:** 2024-07-24

**Authors:** Kasana Raksamani, Manisa Tangvipattanapong, Napat Charoenpithakwong, Suparit Silarat, Natthaphorn Pantisawat, Vutthipong Sanphasitvong, Nattaya Raykateeraroj

**Affiliations:** 1 Department of Anesthesiology, Faculty of Medicine Siriraj Hospital, Mahidol University, 2 Wanglang Road, Bangkok Noi, Bangkok, 10700 Thailand; 2 Department of Anesthesiology, Faculty of Medicine, Khon Kaen University, Khon Kaen, Thailand; 3 Department of Anesthesiology, Lampang Hospital, Lampang, Thailand; 4 Division of Cardio-Thoracic Vascular Surgery, Department of Surgery, Faculty of Medicine Siriraj Hospital, Mahidol University, Bangkok, Thailand

**Keywords:** Acute type a aortic dissection, Postoperative stroke, Surgical risk factors

## Abstract

**Background:**

Despite advances in surgical techniques, the incidence of stroke following acute type A aortic dissection (ATAAD) repair remains markedly high, with substantial immediate and long-term adverse outcomes such as elevated mortality, extended hospital stays, and persistent neurological impairments. The complexity of managing ATAAD extends beyond the operation itself, highlighting a crucial gap in research concerning modifiable preoperative patient conditions and perioperative anesthetic management strategies.

**Objectives:**

This investigation aimed to elucidate the incidence, consequences, and perioperative determinants of stroke following surgical intervention for acute type A aortic dissection (ATAAD).

**Methods:**

In a multicenter retrospective analysis, 516 ATAAD surgery patients were evaluated. The data included demographic information, clinical profiles, surgical modalities, and outcomes. The primary endpoint was postoperative stroke incidence, with hospital mortality and other complications serving as secondary endpoints.

**Results:**

Postoperative stroke occurred in 13.6% of patients (70 out of 516) and was associated with significant extension of the ICU (median 10 vs. 5 days, *P* < 0.001) and hospital stay (median 18 vs. 12 days, *P* < 0.001). The following key independent stroke risk factors were identified: modified Frailty Index (mFI) ≥ 4 (odds ratio [OR]: 4.18, 95% confidence interval [CI]: 1.24–14.1, *P* = 0.021), common carotid artery malperfusion (OR: 3.76, 95% CI: 1.23–11.44, *P* = 0.02), pre-cardiopulmonary bypass (CPB) hypotension (mean arterial pressure ≤ 50 mmHg; OR: 2.17, 95% CI: 1.06–4.44, *P* = 0.035), ≥ 20% intraoperative decrease in cerebral regional oxygen saturation (rSO_2_) (OR: 1.93, 95% CI: 1.02–3.64, *P* = 0.042), and post-CPB vasoactive-inotropic score (VIS) ≥ 10 (OR: 2.24, 95% CI: 1.21–4.14, *P* = 0.01).

**Conclusions:**

Postoperative stroke significantly increases ICU and hospital durations in ATAAD surgery patients. These findings highlight the critical need to identify and mitigate major risks, such as high mFI, common carotid artery malperfusion, pre-CPB hypotension, significant cerebral rSO_2_ reductions, and elevated post-CPB VIS, to improve outcomes and reduce stroke prevalence.

**Trial Registration:**

Thai Clinical Trials Registry (TCTR20230615002). Date registered on June 15, 2023. Retrospectively registered.

## Background

Acute type A aortic dissection (ATAAD) constitutes a significant health emergency that markedly increases the likelihood of stroke after surgical treatment and is associated with high morbidity and mortality rates [1]. The long-term survival of patients with ATAAD, particularly those who develop postoperative strokes, remains uncertain [1]. However, the complication of postoperative stroke following ATAAD substantially escalates hospital and midterm mortality, prolongs stays in intensive care and hospital settings, and elevates the incidence of persistent neurological impairments [1–3]. Despite advancements in surgical and perfusion techniques, the incidence of postoperative stroke following ATAAD repair remains very high, ranging from 13 to 16%, which is significantly greater than the 7–10% incidence associated with other thoracic aortic procedures [1, 2, 4, 5].

A thorough review identified various risk factors for postoperative stroke. These include malperfusion syndrome, involvement of the common carotid artery, unique anatomical variations such as the bovine aortic arch, and certain surgical interventions (most notably total arch replacement) [1, 2, 4]. Additionally, conditions such as cardiogenic shock, pericardial tamponade, cardiac arrest, and malperfusion syndrome—especially cerebral malperfusion—are closely associated with an elevated risk of postoperative stroke [1, 2, 6–8]. However, preoperative neurological symptoms have not been recognized as an independent risk factor for stroke following ATAAD repair [1, 2, 6, 9]. Additionally, the length of surgical procedures, particularly the time spent on cardiopulmonary bypass (CPB) with the aortic cross-clamp in place, has also been identified as a significant contributing factor [2–4]. Nevertheless, there is a notable lack of research focusing on modifiable preoperative patient conditions and perioperative anesthetic management strategies.

Managing perioperative hemodynamics during ATAAD surgery constitutes a notable challenge, particularly maintaining a balance between hemostasis and organ perfusion [10–12]. Ischemic stroke, the primary stroke type associated with ATAAD, often results from complications such as common carotid artery involvement and hypoperfusion [12]. However, the establishment of optimal blood pressure levels during various anesthesia stages—including induction, pre-CPB, CPB, and post-CPB—remains a contentious and insufficiently explored field [13, 14].

Frailty, characterized by a physiological downturn and increased susceptibility, is linked to adverse surgical outcomes [15–17]. The modified Frailty Index (mFI), based on the Canadian Study of Health and Aging Frailty Index [16, 18], is extensively used to assess frailty and predict surgical outcomes in diverse medical domains [16, 19, 20]. Concerning frailty and stroke, a meta-analysis has shown that frail patients who suffer strokes tend to have poorer outcomes, including extended hospital stays, reduced quality of life due to disability, and increased mortality across different follow-up intervals [21]. Although its relevance in open cardiac surgery is well established [17, 22, 23], the impact of frailty on ATAAD patient outcomes has not been thoroughly investigated. Additionally, ATAAD patients who underwent open surgical repair, particularly older individuals prone to frailty and those with preoperative malperfusion, are noted to suffer more postoperative complications and unfavorable outcomes [24].

This study was designed to examine the incidence of stroke following ATAAD surgery, evaluate patient outcomes, and investigate associated risk factors, including preoperative conditions such as frailty, surgical interventions, and perioperative hemodynamic measures. By addressing these crucial aspects, this research intends to contribute valuable insights into the management and prognosis of ATAAD, with the ultimate goal of enhancing patient care in this complex area of cardiovascular treatment.

## Materials and methods

### Study population

Ethical approval was obtained from all participating institutions, and the requirement for informed consent was waived. This multicenter retrospective cohort study was carried out at three tertiary medical centers, enrolling a cohort of 516 patients aged 18 years or older who underwent surgical treatment for ATAAD between 2017 and 2022. All identifying information was removed to ensure confidentiality.

### Definitions

Stroke was defined as the onset of new neurological symptoms resulting from an ischemic or hemorrhagic cerebrovascular event, confirmed through radiological imaging (computed tomography or magnetic resonance imaging) and persisting for more than 24 h. The stroke could occur during surgery or within the first week postoperatively. A permanent neurological deficit was identified by new, lasting impairments in focal or global cerebral function that persisted beyond 24 h and remained until hospital discharge or the patient’s death. Conversely, a transient neurological deficit was defined as postoperative confusion, agitation, delirium, prolonged obtundation, temporary parkinsonism, or neurological symptoms with no evidence on brain computed tomography, all resolving by the time of discharge.

### Data collection

Patient data were meticulously extracted from the electronic medical records of the institutions involved. This study primarily aimed to quantify the incidence of postoperative stroke and other neurological issues, such as delirium, seizures, and spinal cord ischemia. The secondary objective was to assess the outcomes of patients who suffered a stroke and to pinpoint the associated risk factors.

The collection of demographic and clinical data included variables such as age, sex, body mass index, physical status according to the American Society of Anesthesiologists classification, mFI, extent of dissection, indicators of preoperative organ malperfusion, surgical intervention, cannulation sites, duration of operation and CPB, application of deep hypothermic circulatory arrest, transfusion requirements, in-hospital mortality, cardiovascular and respiratory complications, other adverse events, length of stay in the intensive care unit (ICU), and the vasoactive-inotropic score (VIS).

Preoperative neurological status, including symptoms such as altered consciousness, seizures, abnormal movements, or paresthesia, was comprehensively recorded for all participants. Under the standard protocol across all centers in this study, patients displaying neurological impairments before surgery underwent brain imaging via computerized tomography or magnetic resonance imaging along with consultations with neurology specialists for an initial assessment. In the postoperative phase, patients exhibiting neurological symptoms or deficits underwent further evaluations, with neuroimaging employed to ascertain whether these deficits were newly developed or related to preexisting conditions. In every case of neurological symptoms or deficits, whether pre- or postsurgery, thorough assessments by neurology specialists were conducted to guide appropriate management.

Postoperative stroke was identified as the development of novel neurological deficit symptoms, signaling an ischemic or hemorrhagic cerebrovascular event, validated through imaging studies within 7 days postsurgery and enduring over 24 h. Neurological consultations for additional radiological assessments were initiated upon suspicion of postoperative stroke, facilitating the commencement of suitable treatment.

The VIS was calculated as follows: dopamine dose (mcg/kg/min) + dobutamine dose (mcg/kg/min) + 100 × epinephrine dose (mcg/kg/min) + 10 × milrinone dose (mcg/kg/min) + 100 × norepinephrine dose (mcg/kg/min) [25]. Medication dosing rates were meticulously recorded from the pre-CPB phase through the cessation of CPB to the end of the surgical procedure.

The preoperative status of the patients was evaluated using the mFI. This index encompasses 11 criteria, each with a designated score: dependency in daily activities (partial or total), diabetes mellitus (requiring medication), chronic lung disease (chronic obstructive pulmonary disease or recent pulmonary infection within the last 30 days), congestive heart failure within the last 30 days, myocardial infarction in the past 6 months, history of coronary intervention, bypass surgery or angina within the last 30 days, treated hypertension, peripheral vascular disease, recent sensorium impairment, transient ischemic attack or cerebrovascular accident without residual neurological deficit, and cerebrovascular accident with neurological deficit [16, 18].

### Statistical analysis

The calculation of the sample size was performed by a preceding study that documented a 20% incidence of postoperative stroke following ATAAD surgery [1]. With a designated acceptable error margin of 3.5% and a Type I error probability of 0.05, the required sample size was determined to be 502 patients. Given the secondary objective of the study and the application of multiple logistic regression analysis, a rounded sample size of 500 patients was deemed appropriate.

The statistical analysis was performed utilizing IBM SPSS Statistics, version 28 (IBM Corp.). The significance threshold was set at a *P* value of 0.05. Continuous data are depicted as either the mean or median (interquartile range) depending on normality of distribution, whereas categorical data are presented as counts (percentages).

The primary objectives of this study were to assess the incidence of postoperative stroke among ATAAD patients, evaluate patient outcomes, and explore associated modifiable perioperative risk factors. For univariable analysis, chi-square or Fisher’s exact tests were applied to categorical variables, while independent t tests or Mann–Whitney U tests were used for continuous variables, depending on their normal or non-normal distribution. Multivariable logistic regression models were then developed to examine predictors of postoperative stroke using a stepwise approach. The Akaike Information Criterion (AIC) guided the comparison of different models to ascertain the optimal combination of preoperative and intraoperative predictors. With the use of a stepwise approach, variables were incrementally added or removed, each addition or removal refining the model by penalizing added complexity. This process continued until further changes ceased to reduce the AIC value [26]. Ultimately, the model with the lowest AIC value was selected as the most suitable. Variables that achieved a *P* value of less than 0.05 were subsequently included in a multivariable logistic regression analysis to identify factors associated with the occurrence of postoperative stroke, maintaining a *P* value of less than 0.05 as the benchmark for statistical significance.

## Results

**Fig. 1:**
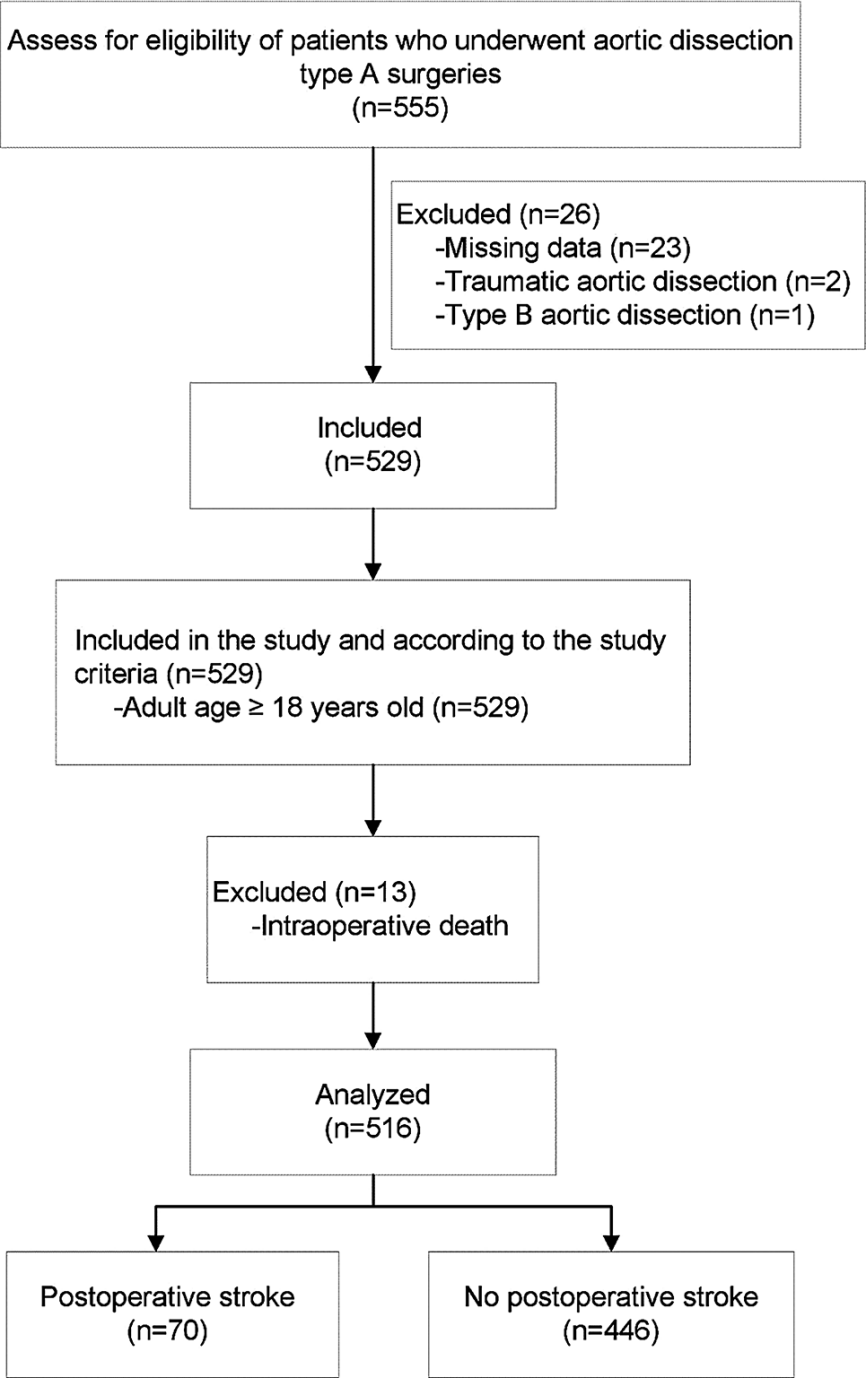
Study flow diagram

The study initially enrolled 555 patients who had undergone aortic dissection surgery. Thirty-nine of these patients were subsequently excluded due to missing data (*n* = 23), traumatic aortic dissection (*n* = 2), type B aortic dissection (*n* = 1), or intraoperative fatalities (*n* = 13), as detailed in Fig. 1. After these exclusions, the study analyzed 516 patients.

Table 1 outlines the demographic and preoperative characteristics of the patients. Of those analyzed, 70 (13.6%) experienced postoperative strokes, whereas 446 (86.4%) did not. The proportion of males was slightly greater in the stroke group (65.7%) than in the nonstroke group (60.1%), but this difference was not statistically significant (*P* = 0.361). The mean age was marginally greater in the stroke group (60.81 ± 12.62 years) than in the nonstroke group (57.52 ± 13.49 years), with a *P* value nearing significance at 0.056. The body mass indices were similar in both cohorts. Patients in the stroke group were predominantly classified as American Society of Anesthesiologists physical status IV. The incidence of emergency surgeries was greater in the stroke group, although the difference was not significant (*P* = 0.068). No significant disparities were observed in complications related to the dissection (such as hemopericardium, hemothorax, or severe aortic regurgitation) between the two groups.

**Table 1:** Demographic and preoperative characteristics of patients. ASA: American Society of Anesthesiologists, BMI: body mass index, CPB: cardiopulmonary bypass, VIS: vasoactive-inotropic score

Variables	Stroke (*n* = 70)	No stroke (*n* = 446)	*P* value
Sex			
Male	46 (65.7)	268 (60.1)	0.361
Age (y)	60.81 ± 12.62	57.52 ± 13.49	0.056
BMI (kg/m^2^)	24.58 ± 4.61	24.31 ± 4.55	0.652
ASA physical status			
III	4 (5.7)	70 (15.7)	0.073
IV	63 (90.0)	364 (81.6)	
V	3 (4.3)	12 (2.7)	
Surgery			
Emergency	65 (92.9)	375 (84.1)	0.068
Urgency	5 (7.1)	71 (15.9)	
Complications of dissection			
Hemopericardium	17 (24.3)	110 (24.7)	1.000
Hemothorax	4 (5.7)	22 (4.9)	0.768
Severe aortic regurgitation	13 (18.6)	97 (21.7)	0.639
Myocardial infarction	3 (4.3)	19 (4.3)	1.000
Organ malperfusion			
Renal	1 (1.4)	9 (2.0)	1.000
Gastrointestinal	0	4 (0.9)	1.000
Central nervous system	7 (10.0)	19 (4.3)	0.069
Limb ischemia	3 (4.3)	29 (6.5)	0.602
Cardiac arrest	0	10 (2.2)	0.372
Extension of dissection			
Aortic root	0	15 (3.4)	0.109
Ascending aorta	10 (14.3)	71 (15.9)	
Aortic arch	10 (14.3)	68 (15.2)	
Descending aorta	19 (27.1)	66 (14.8)	
Abdominal aorta	10 (14.3)	87 (19.5)	
Iliac artery	21 (30.0)	139 (31.2)	
Modified Frailty Index			
< 4 factors	63 (90.0)	430 (96.4)	0.025*
≥ 4 factors	7 (10.0)	16 (3.6)	
Imaging			
Common carotid artery dissection	15 (21.4)	74 (16.6)	0.311
Common carotid artery malperfusion	7 (10.0)	10 (2.2)	0.004*
Thrombus in the aorta	12 (17.1)	76 (17.0)	1.000
Pre-CPB VIS ≥ 10 (*n* = 15)	4 (5.7)	11 (2.5)	0.133

The data are presented as n (%) or mean (SD)

*P* value < 0.05 is considered statistically significant. * Indicated *P* value < 0.05

The mFI revealed that the stroke group had a significantly greater proportion of patients (10%) with four or more preoperative factors than the no-stroke group (3.6%). The prevalence of common carotid artery malperfusion was notably higher in the stroke group (10% of these patients) than in the no-stroke group (2.2%). Furthermore, extension of the dissection into the descending aorta was more common in the stroke group (27.1%) than in the no-stroke group (14.8%).

The analysis of surgical procedures indicated no significant difference between the groups for specific surgeries, such as ascending hemiarch and carotid bypass. However, the stroke group experienced significantly longer median durations for surgery (475 vs. 404 min; *P* < 0.001), CPB (251 vs. 217 min; *P* = 0.003), and deep hypothermic circulatory arrest (49 vs. 42 min; *P* = 0.033).

The frequency of hypotension prior to CPB (mean arterial pressure [MAP] ≤ 50 mmHg) was significantly greater in the stroke group (*P* = 0.006). A substantial reduction in cerebral regional oxygen saturation (rSO_2_) of ≥ 20% from baseline was also significantly more common in the stroke group (*P* = 0.007). Intraoperative blood transfusion volumes and post-CPB VISs exceeding 10 were significantly greater in the stroke group (*P* = 0.010 and *P* = 0.004, respectively), as shown in Table 2.

**Table 2:** Characteristics during intraoperative period

Intraoperative characteristics	Stroke (n = 70)	No stroke (n = 446)	*P* value
Operation			
Ascending hemiarch	34 (48.6)	226 (50.7)	0.798
Carotid bypass	4 (5.7)	23 (5.2)	0.775
Extended root			
Bentall	18 (25.7)	144 (32.3)	0.332
David	1 (1.4)	4 (0.9)	0.519
Wheat	0	2 (0.4)	1.000
Extended arch			
Partial arch replacement	7 (10)	49 (11)	1.000
TAR	10 (14.3)	74 (16.6)	0.729
TAR with FET	17 (24.3)	73 (16.4)	0.126
CABG	13 (18.6)	59 (13.2)	0.264
Aortic valve surgery	6 (8.6)	73 (16.4)	0.108
Arterial cannulation			
Aortic	19 (27.1)	86 (19.3)	0.150
Axillary	40 (57.1)	295 (66.1)	0.177
Femoral	32 (45.7)	186 (41.7)	0.603
Operation time (min)	475 (410–565)	404 (320–480)	< 0.001*
CPB duration (min)	251 (209–297)	217 (167–275)	0.003*
Aortic cross-clamping time (min)	147 (115–191)	132 (109–174)	0.080
DHCA time (n = 426)	49 (36–70)	42 (32–58)	0.033*
No selective cerebral perfusion (n = 7)	2 (3.3)	5 (1.1)	0.207
Selective ACP (n = 407)	58 (82.9)	355 (79.6)	0.630
Selective RCP (n = 12)	2 (2.9)	10 (2.2)	0.671
Anesthetic maintenance			
TIVA	19 (27.1)	109 (24.4)	0.656
Inhalation	51 (72.9)	337 (75.6)	
Dexmedetomidine (n = 37)	3 (4.3)	34 (7.6)	0.455
Steroid (n = 228)	191 (42.8)	37 (52.9)	0.122
Tranexamic acid (mg/kg)	26.6 (16.9–33.3)	25.9 (16.7–33.3)	0.638
Episodes of hypotension (MAP ≤ 50 mmHg, > 5 min)			
Pre-CPB (n = 89)	21 (30.0)	68 (15.2)	0.006*
CPB (n = 383)	53 (75.7)	330 (74.0)	0.883
Post-CPB (n = 82)	14 (20.0)	68 (15.2)	0.296
Any cerebral rSO_2_ drop of ≥ 20% from baseline (n = 208)	40 (57.1)	168 (37.7)	0.007*
Nadir hematocrit	21.5 (19–24)	21.0 (18.9–24)	0.488
Intraoperative blood transfusion			
PRC (ml)	1,650 (1,057–2,350)	1,303 (780–2,000)	0.010*
FFP (ml)	1,168 (472–2,000)	1,072 (528–1,750)	0.479
Platelet (ml)	400 (310–630)	365 (255–610)	0.145
Cryoprecipitate (ml)	220 (80–290)	165 (0–298)	0.339
Post-CPB VIS ≥ 10 (n = 183)	36 (51.4)	147 (33.0)	0.004*

Parametric data are presented as n (%) or mean (SD). Nonparametric data are presented as median (interquartile range)

*P* value < 0.05 is considered statistically significant. * Indicated *P* value < 0.05. ACP: antegrade cerebral perfusion, CABG: coronary artery bypass graft, CPB: cardiopulmonary bypass, DHCA: deep hypothermic circulatory arrest, FET: frozen elephant trunk, FFP: fresh frozen plasma, MAP: mean arterial pressure, PRC: packed red cells, RCP: retrograde cerebral perfusion, rSO_2_: regional oxygen saturation, TAR: total arch replacement, TIVA: total intravenous anesthesia, VIS: vasoactive-inotropic score

Postoperative outcomes, detailed in Table 3, demonstrated a notable increase in neurological complications, such as seizures and delirium, within the stroke group (*P* < 0.001 and *P* = 0.036, respectively). This group also exhibited increased incidences of cardiovascular, respiratory, gastrointestinal, and renal complications, alongside an elevated frequency of reoperations and extended durations of intubation, stays in the ICU, and overall hospitalization (all *P* < 0.001). These findings highlight the substantial impact of stroke on postoperative recovery. Additionally, the hospital mortality rate was marginally greater in the stroke group and approached statistical significance (*P* = 0.050).

**Table 3:** Outcomes of patients in relation to postoperative stroke

Variables	All (*n* = 516)	Stroke (*n* = 70)	No stroke (*n* = 446)	*P* value
Morbidity				
Central nervous complications				
Spinal cord ischemia	12 (2.3)	1 (1.4)	11 (2.5)	1.000
Seizure	38 (7.4)	23 (32.9)	15 (3.4)	< 0.001*
Delirium	85 (16.5)	18 (25.7)	67 (15.0)	0.036*
Cardiovascular complications	195 (37.8)	39 (55.7)	156 (35.0)	0.001*
Respiratory complications	169 (32.8)	35 (50.0)	134 (30.0)	0.001*
Gastrointestinal complications	46 (8.9)	13 (18.6)	33 (7.4)	0.005*
Renal complications	125 (24.2)	30 (42.9)	95 (21.3)	< 0.001*
Re-operation	81 (15.7)	19 (27.1)	62 (13.9)	0.008*
Length of intubation (days)	3 (2-6)	7 (4-16)	3 (2-5)	< 0.001*
Length of ICU stay (days)	5 (3-8)	10 (6-16)	5 (3-7)	< 0.001*
Length of hospital stay (days)	13 (9-21)	18.5 (13-29)	12 (9-20)	< 0.001*
Hospital mortality				
Postoperative death	40 (7.8)	10 (14.3)	30 (6.7)	0.050
Postoperative survival days (*n* = 40)	10 (6-19)	10 (6-14)	10 (5-23)	0.724

Parametric data are presented as n (%) or mean (SD).

Nonparametric data are presented as median (interquartile range)

*P* value < 0.05 is considered statistically significant. * Indicated *P* value < 0.05

ICU: intensive care unit

For the multivariable logistic regression analysis, the AIC was used to identify the best-fitting model for determining predictors of postoperative stroke. The selected model, which achieved the lowest AIC value of 305.9, revealed five key predictors. These were an mFI ≥ 4 (odds ratio [OR]: 4.18, 95% confidence interval [CI]: 1.24–14.1, *P* = 0.021), common carotid artery malperfusion (OR: 3.76, 95% CI: 1.23–11.44, *P* = 0.02), hypotension before CPB (MAP ≤ 50 mmHg; OR: 2.17, 95% CI: 1.06–4.44, *P* = 0.035), a reduction in cerebral rSO_2_ ≥ 20% from baseline (OR: 1.93, 95% CI: 1.02–3.64, *P* = 0.042), and a post-CPB VIS > 10 (OR: 2.24, 95% CI: 1.21–4.14, *P* = 0.01). These factors, corroborated by the univariable analysis, further confirmed their association with the risk of postoperative stroke, as detailed in Table 4.

**Table 4:** Multivariable logistic regression analysis of predictors for postoperative stroke

Variables	Crude OR (95% CI)	*P* value	Adjusted OR (95% CI)	*P* value
mFI ≥ 4 factors	2.99 (1.18–7.54)	0.021	4.18 (1.24–14.1)	0.021*
CCA malperfusion	4.84 (1.78–13.19)	0.002	3.76 (1.23–11.44)	0.020*
Pre-CPB MAP ≤ 50 mmHg	2.38 (1.34–4.22)	0.003	2.17 (1.06–4.44)	0.035*
Cerebral rSO_2_ drop ≥ 20%	1.90 (1.03–3.51)	0.039	1.93 (1.02–3.64)	0.042*
Post-CPB VIS ≥ 10	2.15 (1.30–3.58)	0.003	2.24 (1.21–4.14)	0.010*

*P* value < 0.05 is considered statistically significant. * Indicated *P* value < 0.05

CCA: common carotid artery, CI: confidence interval, CPB: cardiopulmonary bypass, MAP: mean arterial pressure, mFI: modified Frailty Index, OR: odds ratio, rSO_2_: regional oxygen saturation, VIS: vasoactive-inotropic score

## Discussion

In this cohort of 516 individuals who underwent surgery for ATAAD, 13.6% (70) of the patients experienced postoperative stroke, which aligns with the rates reported in previous studies [1, 2, 4]. The diagnosis of these strokes was confirmed through neuroimaging and consultation with neurology specialists. Notably, however, this incidence is substantially higher than that reported in the IRAD study, where the combined incidence of stroke and coma was approximately 6%. Several factors could account for this discrepancy. These include the larger sample size of the IRAD registry and demographic differences in the patient populations. Additionally, variations in surgical techniques, bypass management protocols, and potential disparities in how stroke was defined (which the IRAD study did not detail) may also contribute to the observed differences [27].

A notable trend emerged among ATAAD patients who had dissections extending into the descending aorta or who exhibited central nervous system malperfusion. These patients showed a greater likelihood of experiencing postoperative strokes than those without such extensions. This observation suggests that involvement of the descending aorta or the central nervous system may elevate the risk of significant arterial supply interruptions to the brain and spinal cord, thereby increasing the potential for neurological sequelae such as stroke [1, 3, 6, 24]. Intriguingly, of the 26 patients presenting with preoperative neurological signs, such as seizures or hemiparesis—often reflective of brain hypoperfusion owing to compromised carotid or innominate artery blood flow—only seven experienced postoperative strokes. These seven patients, despite normal preoperative brain imaging results, manifested acute neurological changes after surgery, including altered consciousness or new-onset seizures. Within this subgroup, five patients were diagnosed with hemorrhagic strokes, and two were diagnosed with ischemic infarctions. Conversely, the remaining 19 patients, despite presenting with similar preoperative neurological symptoms, did not develop postoperative strokes, underscoring the complex and variable nature of neurological outcomes following ATAAD surgery.

The associations between perioperative strokes in ATAAD patients and patient outcomes, particularly mortality, have shown variability. Our analysis revealed that the in-hospital mortality rate was notably greater in patients who suffered postoperative strokes (14.3%) than in those who did not (6.7%). Despite a *P* value of 0.05, which does not establish definitive significance, this finding contrasts with the study by Dumfarth et al. [1], which did not observe such a correlation. On the other hand, Chemtob et al. [2] and Ghoreishi et al. [4] identified postoperative stroke as indicative of increased 30-day mortality risk. Furthermore, patients with stroke had prolonged ICU and hospital stays and faced greater risks of cardiovascular, respiratory, gastrointestinal, and renal complications, in addition to a greater likelihood of requiring reoperation, aligning with previous findings [1, 2, 4]. These results underscore the significant ramifications of postoperative stroke on both morbidity and mortality in ATAAD patients, emphasizing the necessity of identifying modifiable risk factors and enhancing anesthetic management strategies to prevent strokes after ATAAD surgery.

Our investigation identified critical links between postoperative stroke and several factors: an mFI of 4 or greater, common carotid artery malperfusion, hypotension before CPB with a MAP of 50 mmHg or less for more than 5 min, a decrease in intraoperative cerebral rSO_2_ of ≥ 20%, and a high post-CPB VIS of 10 or above. Intriguingly, approximately one-third of the patients with ATAAD were identified as frail, which is a lower percentage than that of patients who underwent other types of open-heart surgeries [22, 23]. This discrepancy likely reflects the relatively younger population affected by ATAAD. Despite the lack of a significant impact of frailty on in-hospital morbidity in ATAAD surgery patients, those without frailty markers demonstrated enhanced long-term survival prospects [22, 23, 28]. Our findings highlight the notable association between higher frailty levels and increased risk of postoperative stroke, emphasizing the value of incorporating frailty assessments into the surgical management of ATAAD patients to potentially diminish stroke risk.

Aligned with the findings of multiple studies, our results substantiate the association between common carotid artery malperfusion and postoperative stroke in ATAAD patients [8, 24, 27, 29, 30]. The involvement of the common carotid artery, which signifies cerebral malperfusion, markedly escalates the risk of perioperative stroke [29–31]. Therefore, the adoption of early reperfusion strategies could play a pivotal role in minimizing neurological complications and improving patient outcomes, underscoring the importance of prompt intervention in patients with common carotid artery malperfusion.

Cardiopulmonary bypass blood pressure management has a complex landscape, with studies offering varied conclusions. While certain investigations associate a lower MAP during CPB with an increased risk of stroke [14, 32, 33] and neurological issues such as delirium [34, 35], our findings did not reveal a significant difference in stroke incidence related to varying MAP targets during CPB. This observation challenges the direct link between lower MAP and elevated risk of postoperative stroke [13, 36]. However, our data align with studies indicating that sustained pre-CPB hypotension, defined as MAP less than 50 mmHg for more than 5 min, is a significant predictor of postoperative stroke [32, 37], negatively influencing both morbidity and mortality [38, 39]. Thus, pre-CPB blood pressure should be meticulously optimized, striking a delicate balance to prevent exacerbating the damage to a dissecting aorta with excessively high pressures, while also ensuring that vital organ perfusion is not compromised by overly low pressures.

The randomized controlled trial of Murkin et al. demonstrated the importance of managing decreases in cerebral rSO_2_ during coronary bypass surgery. Specifically, Murkin and colleagues concluded that effective management is essential for reducing prolonged desaturation, shortening ICU stays, and lowering the risk of major organ complications, including stroke [40]. Our study reinforces this viewpoint, demonstrating that a reduction in cerebral rSO_2_ of ≥ 20% from baseline independently predicts adverse neurological outcomes in patients after cardiothoracic surgery [41–43].

At our study’s three centers, the axillary artery was primarily used for arterial cannulation, and selective antegrade cerebral perfusion was the main method employed during hypothermic circulatory arrest. This approach could potentially enhance cerebral perfusion and oxygenation when combined with monitoring of cerebral rSO_2_. However, the lack of a standardized protocol across the institutions for managing rSO_2_ declines during CPB and deep hypothermic circulatory arrest highlights a critical area for improvement. Addressing this could significantly boost patient outcomes. Prompt correction of decreases in cerebral rSO_2_ during surgery is crucial to minimize the risk of postoperative stroke, reinforcing the importance of vigilant monitoring and management of cerebral oxygenation levels in aortic surgery.

In medical research, the criterion for a “high” VIS is not consistently defined. Indices greater than 5.5 have been linked to increased in-hospital mortality and neurological complications [44], while scores greater than 45 are linked to elevated mortality rates one year after surgery [45]. Our analysis revealed a notable link between a postoperative VIS of 10 or more [25] and the incidence of stroke during surgery for ATAAD, highlighting the hemodynamic challenges encountered after CPB. These findings underscore the critical need for precise management of patients after surgery with vasopressors and inotropes. Tailoring dosages to meet specific hemodynamic conditions is crucial in mitigating the risk of cerebral vasoconstriction [46].

This study focused on identifying independent risk factors for postoperative stroke following ATAAD surgery, with the ultimate aim of identifying preventive strategies for this complication. Prior research has highlighted surgical and perfusion techniques that mitigate stroke risk, such as restricting the duration of circulatory arrest to under 30–40 min [3, 7, 8]. In our analysis, the median duration of deep hypothermic circulatory arrest was 49 min in patients who experienced a stroke, compared to 42 min in those who did not. Despite the statistical significance of this finding (*P* = 0.033), this factor was not confirmed as an independent risk factor in multivariable logistic regression modeling. Additionally, our surgical approach primarily involved using the axillary artery for arterial cannulation and antegrade cerebral perfusion. This technique has been associated with a reduced incidence of neurological complications [1, 2, 8, 47]. However, in our study, the stroke rate did not differ significantly based on the employed surgical techniques.

## Limitations

The inherent retrospective nature of our investigation of postoperative strokes in ATAAD surgery narrows the extent of our conclusions. Consideration must be given to the potential influence of incomplete data collection and the omission of particular cases in the interpretation of our outcomes. Moreover, the observational design of this study does not allow for the establishment of direct causation between the observed factors and postoperative strokes. The incidence of deaths during surgery introduces an additional element of complexity and uncertainty to our findings, further complicating the interpretation of our results.

## Conclusions

In the context of ATAAD surgery, 13.6% of patients suffer postoperative strokes, which are associated with increased lengths of mechanical ventilation and extended ICU and hospital stays. It is crucial to identify and address risk factors such as an elevated mFI, common carotid artery malperfusion, pre-CPB hypotension, reduced cerebral rSO_2_, and a high post-CPB VIS. Emphasizing the necessity for comprehensive preoperative evaluations and customized perioperative care, these findings aim to direct future research toward enhancing surgical and anesthetic practices. The ultimate objective is to reduce the occurrence of postoperative strokes and improve patient outcomes in this specialized area of surgical intervention.

## Data Availability

The datasets used and/or analyzed during the current study are available from the corresponding author on reasonable request.

## Abbreviations

ATAAD: acute type A aortic dissection
CPB: Cardiopulmonary bypass
mFI: Modified Frailty Index
ICU: Intensive care unit
VIS: Vasoactive-inotropic score
rSO _2_: Regional oxygen saturation
